# The Challenge of Global Warming in Water Buffalo Farming: Physiological and Behavioral Aspects and Strategies to Face Heat Stress

**DOI:** 10.3390/ani13193103

**Published:** 2023-10-05

**Authors:** Fabio Napolitano, Giuseppe De Rosa, Alfonso Chay-Canul, Adolfo Álvarez-Macías, Alfredo M. F. Pereira, Andrea Bragaglio, Patricia Mora-Medina, Daniela Rodríguez-González, Ricardo García-Herrera, Ismael Hernández-Ávalos, Adriana Domínguez-Oliva, Corrado Pacelli, Emilio Sabia, Alejandro Casas-Alvarado, Brenda Reyes-Sotelo, Ada Braghieri

**Affiliations:** 1 Scuola di Scienze Agrarie, Forestali, Alimentari ed Ambientali, Università Degli Studi della Basilicata, 85100 Potenza, Italycorrado.pacelli@unibas.it (C.P.);; 2 Department of Agricultural Sciences, University of Naples Federico II, 80055 Portici, Italy; giderosa@unina.it; 3 División Académica de Ciencias Agropecuarias, Universidad Juárez Autónoma de Tabasco, Villahermosa 86025, Mexico; 4 Neurophysiology, Behavior and Animal Welfare Assessment, DPAA, Universidad Autónoma Metropolitana (UAM), Mexico City 04960, Mexico; aalvarez@correo.xoc.uam.mx (A.Á.-M.);; 5 Mediterranean Institute for Agriculture, Environment and Development (MED), Institute for Advanced Studies and Research, Universidade de Évora, 7006-554 Évora, Portugal; apereira@uevora.pt; 6 Consiglio per la Ricerca in Agricoltura e l’Analisi Dell’Economia Agraria (CREA), Research Centre for Engineering and Food Processing, Via Milano 43, 24047 Treviglio, Italy; andrea.bragaglio@crea.gov.it; 7 Facultad de Estudios Superiores Cuautitlán, Universidad Nacional Autónoma de México (UNAM), FESC, Ciudad de México 04510, Mexico

**Keywords:** water buffalo, global warming, heat stress, behavior and thermoregulation, infrared thermography

## Abstract

**Simple Summary:**

Global warming (GW) is a current challenge for livestock systems, including water buffalo farms. Buffaloes have anatomical traits such as thick skin and a high density of capillaries and arterioles to improve sensitive heat losses. However, they are exposed to high temperatures and tropical and humid climates that make them susceptible to heat stress. The present review aims to analyze the adverse effects that GW has on the productive performance and health of water buffaloes. The physiological, morphological, and behavioral characteristics of the species will be discussed to understand the impact of GW on buffaloes’ thermoregulation. Additionally, the effectiveness of implementing strategies such as a physical enrichment of the facilities or a thermal assessment through infrared thermography will be discussed.

**Abstract:**

Water buffaloes have morphological and behavioral characteristics for efficient thermoregulation. However, their health, welfare, and productive performance can be affected by GW. The objective of this review was to analyze the adverse effects of GW on the productive behavior and health of water buffaloes. The physiological, morphological, and behavioral characteristics of the species were discussed to understand the impact of climate change and extreme meteorological events on buffaloes’ thermoregulation. In addition, management strategies in buffalo farms, as well as the use of infrared thermography as a method to recognize heat stress in water buffaloes, were addressed. We concluded that heat stress causes a change in energy mobilization to restore animal homeostasis. Preventing hyperthermia limits the physiological, endocrine, and behavioral changes so that they return to thermoneutrality. The use of fans, sprinklers, foggers, and natural sources of water are appropriate additions to current buffalo facilities, and infrared thermography could be used to monitor the thermal states of water buffaloes.

## 1. Introduction

Global warming (GW) refers to the increase in the global temperature since 1880, where the worldwide temperature has increased by an average of 1 °C [1]. Projections to 2080 calculate an expected increase of 3.5–5.5 °C [2]. When addressing GW and livestock species, heat stress is the most important outcome that negatively impacts animals’ health, welfare, and productive performance [3]. Apart from the health issues that it represents to animals, heat stress has serious economic implications by increasing the morbidity and mortality of farm animals and decreasing their feed intake, feed conversion ratio, fertility, and conception rate, as well as causing low milk yields [4,5]. For example, losses of around 63.9% (USD 1.5 billion per year and up to USD 2.26 billion) in the dairy industry of the United States have been associated with heat stress, while financial losses of 60% (up to USD 39.94 billion/year) have been estimated in tropical climates [2,6,7]. In countries like India, a reduction of 0.73 million liters of milk has been attributed to heat stress [8], while milk yield losses reach up to 2.4% in the United Kingdom [9]. Similarly, losses of 15.7% (USD 370 million/year) were reported in the beef industry [7].

Water buffaloes (*Bubalus bubalis*) are known to be heat-tolerant and well-adapted species to humid and tropical climates [10]. However, countries like India, Pakistan, and China—where buffalo farming is prevalent—have reported increases of approximately 0.7 °C, 1.6 °C, and 1.7 °C, respectively, in the past 32 years [11,12,13]. According to different authors, water buffaloes’ thermoneutral zone is considered to be between 13.0 °C and 24 °C, with a relative humidity between 55 and 60% [14,15,16]. When the body temperature of buffaloes exceeds its normal values—ranging from 37.5 °C to 39 °C—a cascade of physiological and behavioral responses are triggered to restore thermoneutrality and prevent heat stress consequences, including health and productive outcomes [17,18].

Water buffaloes are predominant species in humid climates and are adapted to environmental temperatures reaching up to 46 °C [16]. In contrast to *Bos taurus* or *Bos indicus* cattle, buffaloes have morphological characteristics that can represent an advantage/disadvantage in certain climates. For example, high concentrations of melanin in their skin protect buffaloes. The protection provided by melanin is only associated with UVa and UVb radiation. The relationship with solar radiation (essentially short-wavelength radiation) is revealed in a greater absorbance (not necessarily a greater transmittance) and an increase in the skin temperature [16,19]. On the other hand, the skin thickness and the high density of dermal arterioles and capillaries facilitate sensible heat loss through conduction, radiation, and convection [16,17]. The reduced number of functional sweat glands and the sparsely distributed hair might make buffaloes susceptible to heat stress [20,21].

One of the main issues when livestock is exposed to chronic heat stress is the physiological alterations that have negative influences on productive performance. Some authors projected a decrease of 12.78% by 2080 in dairy Murrah buffaloes’ productivity [2]. Moreover, livestock farming can play a non-negligible role in the negative effects of GW due to its direct effect on soil degradation and deforestation attributed to foraging. Another relevant aspect is the contribution of livestock to greenhouse gas (GHG) emissions due to their enteric fermentation, representing 11% of the total emissions. Therefore, promoting sustainable intensive and extensive productive systems and intensive rotational grazing (e.g., holistic systems) is essential for both humans and animals to reduce the adverse influence of farming on GW [22,23,24].

To prevent heat stress consequences and reductions in productive parameters, current buffalo farms have implemented alternatives to promote heat loss via evaporation such as using sprinklers, fans, and natural body waters so buffaloes can perform wallowing—a thermoregulatory species-specific behavior—as well as providing naturally or artificially shaded areas [16]. Furthermore, strategies to alleviate heat stress include methods to recognize thermal alterations in buffaloes, such as the use of infrared thermography (IRT) to detect changes in the surface temperature of animals in response to hyperthermia [25].

Nonetheless, the available information regarding the application of IRT and the effect of heat stress on buffaloes is still developing. Therefore, the present review aims to analyze the adverse effects that GW represents on the productive performance and health of water buffaloes. The physiological, morphological, and behavioral characteristics of the species will be discussed to understand the impact of GW on buffaloes’ thermoregulation. Moreover, the current strategies adopted in buffalo farms (e.g., shaded areas and sprinkler use) will also be described, as well as the use of IRT as a method to counteract the effects of GW.

## 2. Adverse Effects of Global Warming on Livestock Farming: An Approach for Meat and Dairy Products

Currently, more than 8 billion people have increased the demand for meat and dairy products by approximately 40 and 50%, respectively, in the past years [26,27,28,29]. When considering the increase in demand for livestock farming, GW and its projected increases in temperature of 3.5 to 5.5 °C by 2080 represent challenges due to the effects on the productive and reproductive performance of livestock, including buffalo farms [2,27,30].

Buffaloes’ performance, health, and welfare are affected both directly and indirectly by the environment. Although water buffaloes are considered animals that possess morphological, anatomical, and behavioral characteristics that help them adapt to hot and humid climates, heat stress has a detrimental effect on the animals’ productive and reproductive performances. Wolfenson et al. [4] reported that heat stress could cause economic losses of 60% in bovine dairy farms located in the tropics, having effects at the neurophysiological level, reducing fertility due to poor estrus expression, low progesterone secretion, impaired oocyte quality, and low embryonic development, and decreasing the percentage of conception rate from 20 to 27% during the summer [5].

**Figure 1:**
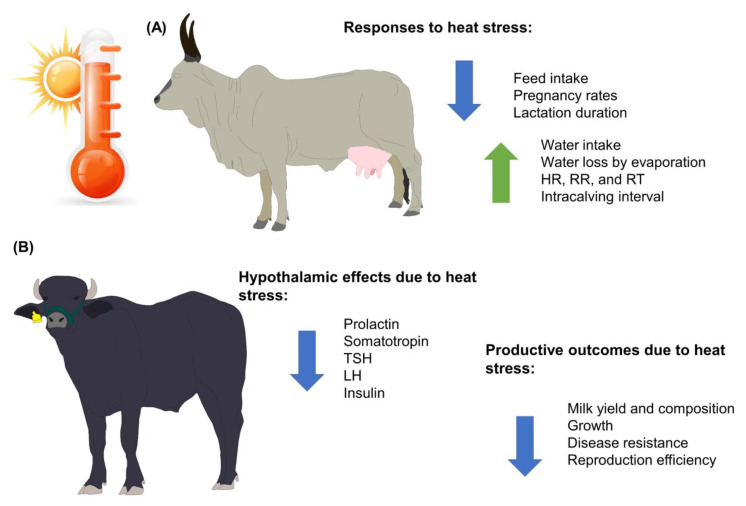
Main effects of heat stress on water buffaloes and bovines of the genus *Bos*. (**A**) Animals from the *Bos* genus. (**B**) Animals from the *Bubalus* genus. HR: heart rate; LH: luteinizing hormone; RR: respiratory rate; RT: rectal temperature; TSH: thyroid-stimulating hormone.

Heat stress in water buffaloes affects milk performance and animal welfare because heat stress decreases feed intake, which is closely linked to the amount of milk that is produced. However, feed intake causes metabolic heat generation, which can be detrimental when animals are already in a heat stress state (Figure 1). In addition, the intensification and confinement of water buffaloes to maintain greater control over their feeding and to achieve better milk yield and constituents have promoted the use of supplementary cooling systems such as fans and foggers to minimize the effects of heat stress on dairy buffaloes in hot weather [31].

To maintain adequate microclimate parameters inside buffalo farming facilities, the room temperature, relative humidity, lighting, and ventilation, among other factors, must be considered [32]. This is relevant because it is estimated that 10–30% of animal productivity is determined by the microclimate [33]. In the case of water buffaloes reared in intensive systems, facilities must promote the thermal comfort of animals by keeping room temperatures between 13.0 °C and 24 °C and keeping humidity percentages between 55 and 60% [14,15,16]. Therefore, adopting strategies to achieve thermoneutrality in animals is important in buffalo farming.

In this sense, foggers distribute air droplets that quickly evaporate and cool the circulating air, increasing convective heat loss under stress conditions in cattle [34]. Seerapu et al. [34] evaluated the effect of microclimate alteration on milk production and milk composition in 40 Murrah buffaloes. By comparing the use of foggers, fans, foggers plus fans, and control conditions, the authors found differences in the rectal temperature (RT) of the buffaloes. While the RT of the control group remained at 39.16 °C, the foggers plus fans group had an RT of 38.61 °C. Similarly, the respiratory rate (RR) and pulse rate decreased, observing a decrease of 15.66 breaths/min and 16.47 beats/min, respectively, in the foggers group. Additionally, the milk yield increased in the foggers plus fans group (+1.26 kg/buffalo/day), improving the nutritional composition of the milk by increasing the percentage of solids-not-fat by +1.08% in the foggers group and by 0.91% in the foggers plus fans group.

Authors such as Das et al. [31] have also evaluated the effect of microclimate modification by providing ceiling fans and mist fans in the shed to 21 Nili-Ravi buffaloes and keeping the same number of buffaloes in separate sheds without modifications in the microclimate and management. Parameters such as the RT, RR, pulse rate, serum cortisol levels, and daily milk yield were evaluated. The results showed that the animals housed in the sheds with ceiling fans and mist significantly increased their milk yield by 17.4%, with improvements in the percentages of fat (+7.71%) and solids-not-fat. Physiologically, in the same group, the RT, pulse rate, and RR decreased by 0.89 °C, 6.48 beats/min, and 4.17 times/min, respectively.

Strategies are required to minimize the consequences of the effects of climate change on buffaloes’ milk yields. For example, Sigdel et al. [35] evaluated the effects of extreme temperatures and relative humidity on the milk yield of Murrah buffaloes. A strong negative correlation (*p* > 0.01) was found between the milk yield and the temperature and humidity index (THI), indicating that the milk yield decreases at the same time that the THI increases (r = −0.80).

The estimated decline in milk yield in buffaloes due to GW ranges from 10 to 30% during the first lactation and from 5 to 20% during the second and third lactations [27]. In addition, GW also affects the quality and quantity of available grass for foraging, and causes an increasing incidence of diseases and reduced water availability that negatively impacts dairy farms [36].

Regarding meat production, water buffaloes are adapted to different production systems because they can efficiently use more highly lignified pastures, with an estimate meat production of 4,290,212 tones, particularly in Asia [37]. Notwithstanding this, Bragaglio et al. [38] compared different diets and found a higher share of protein and fat in the milk of buffaloes (Mediterranean buffalo in Italy) fed with corn silage. Meat buffaloes can be housed on irregular terrain and wetlands, representing a lower environmental impact due to their use in systems that could hardly be used for agriculture or dwellings [39,40].

Grazing buffaloes in grasslands improves soil functionality by preventing desertification and using their manure without increasing methane production. Moreover, buffaloes have more efficient fiber digestion than Bos cattle, making it more suitable for them to consume tropical forages [39]. Although the meat quality highly depends on the buffalo breed and age at slaughter (where young male buffalo meat is considered more suitable [41]), Di Stasio and Brugiapaglia [37] mention that the great capacity of buffaloes to adapt to several rearing areas and management systems is an advantage of buffalo farming. For example, in the Philippines, events such as typhoons, floods, and droughts have influenced the decision of farmers to implement buffalo farming as a sustainable and viable adaptation to climate change [42]. Nonetheless, physiologically and anatomically, buffaloes might have some traits that can make them susceptible to GW; the characteristics will be discussed in the following sections.

## 3. Thermoregulatory Physiology of Water Buffaloes Facing Heat Stress

### 3.1. Physiological Aspects

Thermoregulation aims to maintain body temperature within a certain range where cellular functionality can be preserved. Under normal circumstances, the organism uses a series of physiological, endocrine, and behavioral adaptations to promote thermoneutrality [3]. However, when animals are exposed to hot climates and develop heat stress, the central nervous system (CNS) triggers several responses to restore homeostasis [43,44].

The preoptic area (POA) of the hypothalamus, a key structure for thermoregulation, contains approximately 30–40% of warm-sensitive neurons that respond to environmental warming through peripheral thermoreceptors located in the skin [45,46]. Transient receptor potential cation channels—known as TRP—are a large family of thermosensitive receptors that can detect both heat and cold stimuli [47,48]. Five of these receptors (TRPV1-4 and TRPM2) are activated with non-noxious and noxious heat. TRPV1 and TRPV2 respond to high temperatures between 43 °C and 55 °C, respectively [47], and are expressed in Aδ and C fibers [49].

When considering that heat stress can increase the RT to 39.71 °C in water buffaloes exposed to environmental temperatures around 27–35 °C [17], the activation of these warm-sensitive afferent fibers is essential to initiate a cascade of responses to prevent hyperthermia. Several studies in young and adult buffaloes indicate that environmental characteristics such as the ambient temperature or the facility design affect the internal temperature of water buffalo, potentially reaching critical points. For example, male Murrah calves that were exposed to an open stable without shade and in a humid tropical climate reached body temperatures between 38.5 ± 0.37 °C and 40.5 ± 0.10 °C during the middle of the day [50], while non-lactating and non-pregnant Murrah females that were exposed to non-shaded paddocks reached an RT of up to 39.0 °C [51].

**Figure 2:**
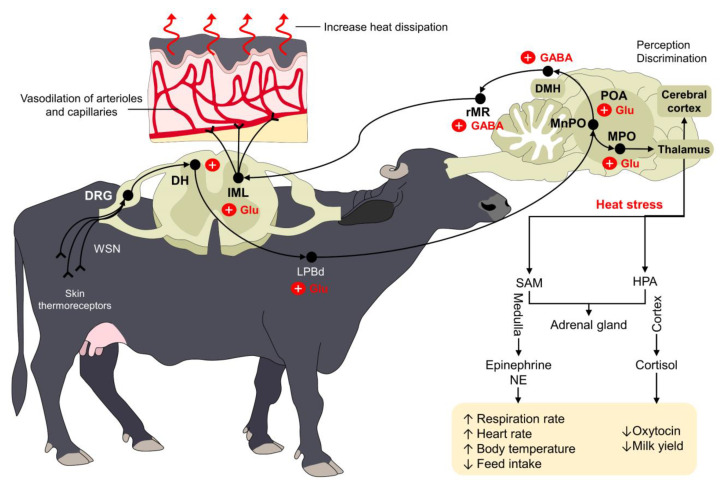
Thermoregulatory pathway of water buffaloes when facing heat stress. Peripheral thermoreceptors process and transmit information about the external thermal environment of mammals. Through a complex connection between the spinal cord (DRG and DH), the LPBd, and supraspinal structures (primarily the POA, MPO, and MnPO), the organism triggers different responses to increase heat dissipation. For example, the vasodilation of dermal arterioles and capillaries increases heat loss in water buffaloes exposed to heat stress. Similarly, other compensatory mechanisms such as increases in respiratory rate and heart rate serve to restore thermoneutrality. DH: dorsal horn; DMH: dorsomedial hypothalamus; DRG: dorsal root ganglion; GABA: gamma amino butyric acid; GLU: glutamate; HPA: hypothalamic–pituitary–adrenal; IML: intermediolateral; LPBd: dorsal part of the lateral parabrachial nucleus; MnPO: median preoptic nucleus; MPO: medial preoptic area; NE: norepinephrine; POA: preoptic area; rMR: rostral medullary raphe region; SAM: sympathetic-adrenomedullary; ↑: increase; ↓: decrease.

The hypothalamus can recognize such increases in the body temperature, and cutaneous thermosensitive receptors can also send excitatory signals to warm-sensitive neurons (WSNs) in the dorsal root ganglion (DRG) of the spinal cord [52]. From the spinal cord, the signal is transmitted to the POA using the dorsal part of the lateral parabrachial nucleus (LPBd), where glutamatergic neurons participate [53]. Other regions of the POA, such as the medial preoptic area (MPOA) and the median preoptic nucleus (MnPO), are also involved in the physiological control against heat stress [44,46]. From these nuclei, efferent pathways leading to skin vasodilation are triggered by projections from the POA to the rostral raphe pallidus (rRPA) [44], while evaporative heat loss mechanisms (e.g., sweating, panting, or tachypnea) use projections from the intermediolateral column cells (IML) in the spinal cord and the rostral ventromedial medulla (RVMM) [54]. This neural pathway is schematized in Figure 2.

The vasodilation of dermal blood vessels is one of the initial resources that animals use to lose heat [21]. A way to assess the amount of dissipated heat from the skin or the level of heat exchange through the skin is by evaluating the surface temperature using IRT devices, as shown by Pereira et al. [17]. In this study, the authors compared the tail and coat surface temperature of female Mediterranean buffaloes raised at two different temperature ranges: a group in 24–27 °C, and another group with higher temperatures between 27 and 35 °C. The results showed that higher environmental temperatures increased the tail (32.83–35.13 °C vs. 34.15–37.96 °C, respectively), and coat temperatures (30.22–34.28 °C vs. 33.20–38.94 °C, respectively). Similarly, Rodríguez-González et al. [55] registered increases of 5 °C in the superficial temperature of the facial and body regions of Buffalypso buffaloes transported under ambient temperatures reaching up to 30 °C. These results reflect a high vasodilatation of epidermal blood vessels to dissipate heat and reduce body temperature.

Although peripheral vasodilation highly modulates heat loss to prevent hyperthermia, it also causes compensatory tachycardia and tachypnea in buffaloes, which also serve as evaporative heat loss mechanisms [14,43]. The studies conducted by Silva et al. [51] reported that female Murrah buffaloes raised in non-shaded paddocks had higher respiratory movements per minute (mov/min) (29.2–34.4 mov/min) than buffaloes with access to shade (28.5–32.6 mov/min). Likewise, Mediterranean buffaloes raised at 27–35 °C registered a high RR (from 26 to 87 mov/min) [17], while water buffaloes’ RR of 95.8 breaths per minute (bpm) were positively correlated with the RT (39.5 °C) (r = 0630) [56].

Regularly, mammals use sensible routes of heat loss, such as conduction, convection, and radiation, to maintain their body temperatures within normal ranges [43]. However, the presented results suggest that water buffaloes recur to vasodilation and evaporative heat loss pathways more intensely and for longer periods when comparing *Bos taurus* cattle and water buffaloes. This has been shown in studies where buffaloes have a higher frequency of heat stress signs such as panting (+29%), tongue protrusion (+27%), and neck extension (+27%) than *Bos* cattle [17,56].

In summary, GW and an increase in the ambient temperature force buffaloes to trigger physiological responses such as tachycardia and tachypnea to dissipate heat. Additionally, the vasomotor changes coordinated by the ANS serve as mechanisms to thermoregulate and prevent heat stress. Nonetheless, the particularities of thermoregulatory responses in water buffaloes are highly influenced by their anatomical characteristics at a dermal level, making them more resistant to cold environments than *Bos* cattle but susceptible to heat stress, as will be described below. These could be helpful in countries like India, where buffalo farming is prevalent with an approximate contribution of 35% to Asia’s milk production because buffaloes are regarded as better adapted to harsh environments.

### 3.2. Anatomical Aspects

**Figure 3:**
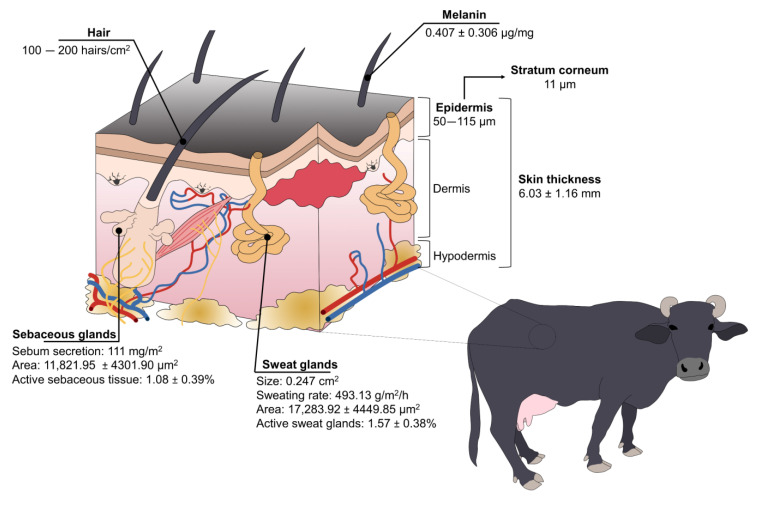
Structure and species-specific characteristics of water buffaloes’ skin.

It is known that, compared to *Bos taurus* and *indicus*, water buffaloes trigger an intense physiological response to direct solar radiation due to anatomical traits such as the color and thickness of the epidermis, hair density, and number of sweat glands (Figure 3) [17,25]. The skin structure has a key role in buffaloes’ thermoregulation due to the sympathetic innervation (including thermoreceptors) that perceives and transmits stimulus to the POA [44].

Buffaloes have a high concentration of melanin in the basal cells of the skin and hair [21,57], with an average of 0.407 ± 0.306 µg/mg and 2.734 ± 2.409 µg/mg, respectively, in Taiwanese water buffaloes [58]. While the color of their skin protects them from ultraviolet radiation—and potentially skin tumors—in tropical regions, it also makes them more susceptible to heat stress because dark-colored skin/coats absorb higher concentrations of radiation [16,19]. Nonetheless, buffaloes have other anatomical adaptations to compensate for this susceptibility.

The skin thickness of buffaloes greatly differs when compared to cattle. Vilela et al. [15] determined that buffaloes’ skin thickness is 6.03 ± 1.16 mm. It is known that the thickness of the epidermis (50–115 µm) represents 1.5–2% of total skin [57], with a stratum corneum of 11 µm [25,59,60], while cattle have 51 µm and 5 µm of thickness, respectively [57]. From a thermoregulatory perspective, this suggests that the thick skin of water buffaloes acts as a thermal insulator against hot air [57]. Moreover, buffaloes have a high density of arterioles and capillaries in the skin, improving the sensible heat loss by conduction, radiation, and convection [16,17].

Improving heat dissipation through skin blood vessels is also associated with their sparsely distributed hair [17]. Contrary to *Bos* genus having approximately 1000 hairs/cm^2^, water buffaloes have 100–200 hairs/cm^2^ [57]. Female Murrah buffaloes have an average hair density of 2.0 ± 0.26 mm^2^ [15]. While some authors mention that this characteristic makes them susceptible to solar radiation absorption [17], Presicce [57] refers to the low density of hair as a trait that facilitates heat loss via convection and radiation, preventing heat stress.

Water buffaloes also have a reduced density of sweat glands because these are highly correlated with the number of hair follicles [21]. Sweat glands were characterized in Murrah buffaloes, finding that the height of the sweat glands, the area, and the active sweat gland tissue was 16.2 ± 1.99 μm, 17,283.92 ± 4449.85 μm^2^, and 1.57 ± 0.38%, respectively [15]. It is said that buffaloes have a less efficient evaporative mechanism to dissipate heat due to their reduced sweating ability [16]. Nonetheless, buffaloes’ sweat glands are twice the size of the *Bos* species (0.247 cm^2^ vs. 0.124 cm^2^), possibly compensating for the lack of functional glands [57]. Furthermore, the sweating rate can be modified according to the environmental temperature, as shown in a study where Mediterranean buffaloes that were exposed to heat stress increased their sweating rate (control: 320.18 g/m^2^/h vs. heat stress: 493.13 g/m^2^/h) [17], similar to the rate of 463 g/m^2^/h in buffaloes under direct solar radiation [50] to increase evaporative heat loss.

Among other skin traits, Marai and Haeeb [16] mention that buffaloes have sebaceous glands with a greater secretion activity than cattle, secreting sebum that can lubricate the skin and prevent water absorption from the skin, helping them to stay cool. A sebaceous gland area of 11,821.95 ± 4301.90 μm^2^ and an active sebaceous gland tissue of 1.08 ± 0.39% were reported in Murrah buffaloes [16]. These values can be associated with the functionality of the glands. In this regard, Shafie and Abou El-Kahir [61] compared the sebum secretion between buffaloes vs. Egyptian, Shorthorn, and Friesian cattle, and found that buffaloes secreted an average of 111 mg/m^2^ of sebum, while cattle had an average of 32 mg/m^2^. However, when comparing the volume of glandular tissue per unit area of skin, buffaloes had a smaller volume. This might suggest that the number of buffaloes’ sebaceous glands is reduced, but they are more efficient in comparison to cattle.

All of these physiological and anatomical traits are aimed to reduce or prevent disruptions such as metabolic disturbances, oxidative stress, and immunosuppression when animals are exposed to heat stress [3]. Buffaloes’ higher ability to achieve sensible heat losses through the network of skin blood vessels, dark skin that improves heat loss via convection and radiation, and sweat glands that are double the size of those found in *Bos* cattle can be considered as adaptative benefits of the species [17]. However, apart from these changes, water buffaloes also resort to species-specific behaviors to maintain thermostability. Water buffaloes frequently engage in wallowing to reduce their body temperatures and minimize the effect of direct solar radiation. Seeking shaded areas (whether natural or artificial) also mitigates heat stress and contributes, together with the physiological and anatomical mechanisms, to reaching thermoneutrality.

## 4. Behavioral Responses of Water Buffaloes to Diminish Heat Stress

### 4.1. Water Immersion

When animals are outside of their thermoneutral zone, which is defined as the environmental temperature range in which the species is in thermal comfort, a series of adaptive thermoregulatory mechanisms are triggered to promote heat loss and maintain the balance between the amount of produced heat via metabolic processes with the one that must be dissipated [21,62,63,64,65,66]. This is relevant because when animals are not able to dissipate heat to reduce their core temperatures, this results in a decreased food intake, low weight gain, and even fertility issues [67]. Within the behavioral responses that water buffaloes use to dissipate heat, water immersion can be listed. When buffaloes have free access to water through ponds, puddles, or potholes, they tend to spend a large part of the day wallowing, dedicating up to 2.35 h a day to this activity [66,68]. This behavior, together with physiological responses such as peripheral vasodilation, decreases the surface temperature of the buffalo via evaporation facilitated through the wet skin [64].

This phenomenon has been studied by Maykel et al. [66], who compared the influence of moderate vs. intense heat on the thermoregulatory behaviors and feeding changes of buffalo heifers in a silvopastoral system and a conventional system in Cuba. The authors found that the activity of immersing themselves in a pond of water was more frequent in buffaloes exposed to intense heat (>35 °C), spending an average of 4.06 h performing this activity, while buffaloes exposed to moderate heat spent 2.91 h inside the water.

Similarly, this immersion activity is favorable for bovine thermolysis, as shown by Mota-Rojas et al. [69] in a study applying the IRT technique to assess the surface temperature of river buffaloes before, during, and after wallowing during the hottest hour of the day (38 °C). Assessing the surface temperature of the thoracoabdominal region of the buffalo showed a decrease of 4 °C while wallowing. After leaving the pond, the buffaloes’ temperature was 32.8 °C in contrast to the 36.4 °C temperature that was registered before they entered the water. This study shows that wallowing helps to dissipate heat. Therefore, the effects of GW such as scarcity of water are a challenge that buffalo farms need to address. However, as mentioned by Napolitano et al. [68], wallowing in the mud contributes to thermoregulation through peripheral vasodilatation and the evaporation of heat, and the layer of mud in the skin might serve as protection against direct radiation [64]. Although wallowing could be considered the main thermoregulatory behavior of water buffaloes, when they are not provided by body water, other behaviors are triggered, such as seeking shaded places.

### 4.2. Seeking Shade

Another behavior that has been observed in buffaloes is seeking shaded areas, whether natural or artificial. When exposed to heat stress, water buffaloes tend to search for shaded areas that help them reduce their RT [70]. Moreover, the feed intake is influenced by the presence of shaded areas since it was reported that buffaloes prefer to graze in temperate climates while reducing foraging and rumination when exposed to heat stress [63,71]. Similarly, ruminants reduce or change their food intake under extreme heat, as shown by Fundora and Sánchez’s [72] study involving 32 buffaloes, which are animals that prefer to graze during night hours or during the freshest hours of the day. The authors concluded that more than 90% of the animals grazed at 7 a.m., while resting hours and the number of ruminations was performed during the hottest time of the day, expending 4.5 h ruminating and 76% of the time resting.

**Figure 4:**
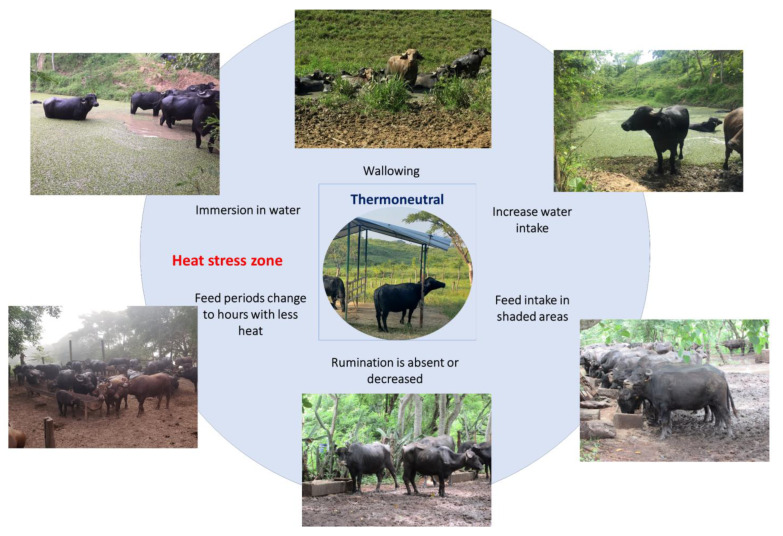
Thermoregulatory behaviors of water buffaloes when exceeding their thermoneutral zone. When the thermoneutral zone of the animals is exceeded and they begin a process of thermal discomfort, they present a series of behaviors to increase heat dissipation. For water buffaloes, the most common thermoregulatory behaviors are immersing themselves in flood zones, wallowing in mud, searching for shaded areas, and modifying their feed intake schedules or reducing their feed intake.

Contrarily to food intake, water consumption increases, and other postural changes such as stretching body extremities are present as ways to dissipate heat [73]. In this sense, decreasing the time spent lying down and standing up exposes a greater amount of skin to air currents, maximizing evaporation [65]. This behavior can increase by up to 10% when heat loads above 15% are present, as mentioned by Tucker et al. [74]. Thermoregulatory behaviors are schematized in Figure 4 [20,65,73,75].

It should be noted that depending on individual adaptability, river buffaloes can withstand higher environmental temperatures without activating their thermoregulation mechanisms [21]. Also, current trends in buffalo farming include the use of natural or artificial shade, pools of water, and resting spaces to promote the thermal comfort of the species.

In summary, water buffaloes resort to thermoregulatory behaviors such as wallowing and seeking shade, together with physiological responses (e.g., vasomotor changes and cardiorespiratory modulation) to decrease body temperature. However, to perform said behaviors, it is important to consider the type of production system (extensive and intensive) and its design to decide if the natural conditions of the environment require modifications such as artificial ventilation or other strategies to promote welfare.

## 5. Influence of Natural/Artificial Shade on Buffaloes’ Thermoregulation

Farm facility improvement to reduce thermal sensation is considered a passive cooling design, where the use of the natural surroundings and the climate, without electronic devices, results in friendly and sustainable options for buffalo farms. Shed designs with particular attention to indoor ventilation, pre-cooled air entering the building, and envelope designs have been shown to reduce the indoor temperature by more than 5 °C in cattle farms [76].

Other techniques to promote normothermia include the use of wallowing areas or showers during summer [16], where water as a resource for heat dissipation facilitates heat exchange between the animal and the environment [21,77]. Silvopastoral systems (pastures combined with trees) are efficient alternatives in the tropics to reduce the effects of heat stress compared to conventional systems (only pastures). This was studied by Galloso-Hernández et al. [66], who evaluated the behavior of buffaloes under both types of production systems between May (intense heat stress) and November (moderate heat stress), both with access to a wallowing area. The results showed that the temperature was 2 °C lower in the silvopastoral system during intense heat stress and that behaviors such as grazing were significantly higher (7.49 vs. 5.96 h) during periods of intense heat stress. In the conventional system, the animals spent more time wallowing and stayed under the shade for longer when exposed to intense heat conditions (2.62 h vs. 1.71 h). These results suggest that silvopastoral systems might be more suitable for water buffaloes under intense heat stress [66], as long as animals are provided shade and wallowing areas that can help them thermoregulate [73].

**Figure 5:**
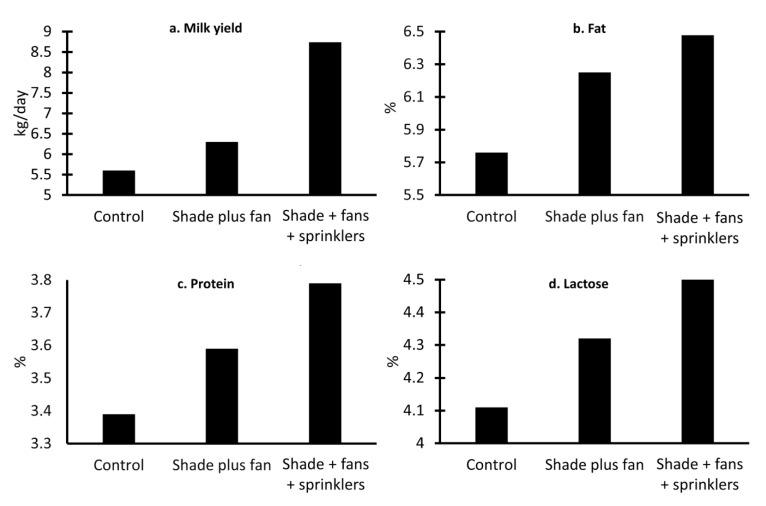
Productive performance of Nili Ravi buffaloes under different ambient management interventions. (**a**) kg of milk produced per day under different conditions: shade (control); shade and fan; and shade plus fan plus sprinklers. An increase of 0.7 kg/day was observed when implementing shade plus fans, while an increase of 3.14 kg/day was reported when using the three elements. (**b**) Percentage of fat in buffalo milk. It was observed that the use of three resources increased milk fat by 0.74%. (**c**) Percentage of protein in buffalo milk. The control group presents 3.39% protein, 0.2%, and 0.4% less than those shown by other groups. (**d**) Percentage of lactose in buffalo milk. In the same way, the control group shows the lowest percentages of lactose (4.11%), followed by the group with shade and fan, with an increase of 0.21%, and the highest values on shade plus fan plus sprinklers.

On the other hand, Younas et al. [78] studied the physiological effect of artificial shade and other elements (e.g., ceiling fans and showers plus ceiling fans) on serological and thyroid hormones in Nili-Ravi buffaloes. The results showed that the use of showers and fans significantly decreased the skin temperature (decreases of up to 2.2%) (*p* < 0.05), RR (breaths per minute decreased by 17%), and pulse (decreases of up to 13.77%) over the other groups. Likewise, this group expressed the highest values in the thyroid analysis, which indicates a normal function of the thyroid gland, a function that can be affected by heat stress [78]. The milk yield is another parameter that can be affected by heat stress, and decreases have been reported, as shown in Figure 5, where the effect of implementing shade, fans, or sprinklers is schematized using data from the study by Ahmad et al. [79]. Using these tools, an increase of 0.7 to 3.34 kg of milk per day was observed, while a tendency to increase the percentages of fat, protein, and lactose was found in buffaloes housed in facilities with more than two resources (shade + fan + sprinkler) due to a higher intake of dry matter [79].

**Figure 6:**
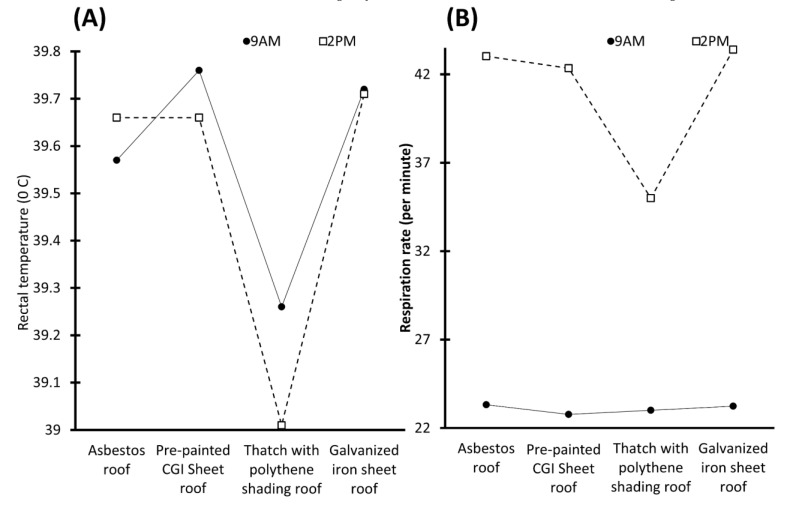
Variation of rectal temperature and respirations per minute in buffalo calves at 9 A.M. and 2 P.M. under different types of roof materials. (**A**) Rectal temperature of calves. It is shown that all the roofs, except for the asbestos roof, have the highest temperature at 2 P.M, with a difference of up to 0.25 °C compared to that presented at 9 A.M. Likewise, it is observed that the buffaloes under the pre-painted CGI sheet roof and galvanized iron sheet roof have the highest temperatures in both hours, while the lowest temperature is recorded in buffaloes under thatch with polythene shading roof, with a difference of 0.5.°C in comparison with the pre-painted CGI sheet roof. (**B**) Breaths per minute. Observe the difference in respirations shown at different times of the day. Thatch with polythene shading roof recorded 8.4 breaths per minute, which is less than those expressed with the galvanized iron sheet roof at 2 P.M.

Although access to water contributes to homeothermy, in places with water scarcity, this could represent a disadvantage. However, it was shown that providing shade can contribute to a reduction of 30% in radiated heat [16,20,80]. This can be reflected in a lower RR, plasma cortisol, heart pulse, and RT in animals provided with shaded areas. This was reported by Kumar et al. [81], who assessed the effect of housing modification on Murrah buffaloes. Two different housing conditions were compared: buffaloes under a loose housing system and buffaloes that were assigned to a shed with an asbestos roof that was 10–12 feet high, provided with sand bedding and ceiling fans. The results indicated that the buffaloes assigned under the open system had significantly higher RT (38.26 vs. 38.64 °C) and skin temperature values (35.10 vs. 33.89 °C) during autumn. Likewise, in contrast to the loose system, the buffaloes in the shed with the ceiling fans had lower RR (37.39 vs. 30.99 counts/min), pulse rate (60.91 vs. 52.52 counts/min), and cortisol levels (4.94 vs. 3.31 ng/mL), which could indicate that the use of sheds, roofs, sand bedding, and fans can decrease physiological reactions to heat stress. Moreover, it is important to consider that the use of asbestos is controversial due to its association with cancer. Reports indicate that commonly used roofing materials include a galvanized iron sheet [82] and thatched roofing [83,84]. Nonetheless, studies show significant differences between shade materials. Barman et al. [85], compared the RR and RT values of 24 buffalo calves housed in different roofs: an asbestos roof (AR); pre-painted CGI sheet roof (PSR); thatch with polythene shading roof (TPS); and galvanized iron sheet roof (GIS). The results indicated that the animals under the TPS roof had a lower RT, while the animals under the GIS and AR had significant increases in the RT. Similarly, a lower RR was recorded in TPS buffalo calves (*p* < 0.05). Based on the relationship between the RR and the level of discomfort, these results suggest that TPS is the most efficient material to provide a favorable micro-environment. These findings by Barman et al. [85] are summarized in Figure 6.

Additionally, apart from considering the thermal benefits that the facility design must provide to animals, as shown in Barman et al.’s [85] study, it is necessary to highlight the possible influence of these materials on animal health. For example, an asbestos roof shed is considered a traditional roof type. However, several health issues such as pulmonary diseases, carcinogenesis, and fibrogenesis have been found in both humans and animals [86,87]. Additionally, current thermocomfortable housing (e.g., thatch roof shed) provide higher benefits for farm animals—by reducing their RT under heat stress—than an asbestos roof [88,89], suggesting that a conventional roof made with asbestos could easily be replaced by other materials to avoid health risks and improve farm designs. In this way, the combination of different shading materials needs to be considered when designing buffalo farm facilities to reduce the THI inside the stables and promote thermal comfort (Table 1).

## 6. Thermal Imaging Applied to Evaluate Buffaloes’ Thermal States

IRT is a tool that is currently widely used in several disciplines such as engineering, construction, and both human and animal medicine [94]. It assesses the surface temperature of the skin as the vasomotor response of the organism to dissipate or conserve heat depending on the thermal state of the animals (i.e., hyperthermia or hypothermia) [95]. Currently, IRT is being applied in veterinary medicine as a real-time method to evaluate hemodynamic changes and the thermostability of animals due to thermal stress, inflammatory processes, pain, stressor exposure, and during different conditions and events (e.g., newborns or during transport) in a wide variety of species [14,55,95,96,97,98,99].

For example, pigs are a species highly susceptible to heat stress due to transport or unloading due to their large deposits of adipose tissue and their scarce distribution of sweat glands. In pigs, IRT has been used to identify thermal imbalances in different thermal windows such as the nasal, ocular, auricular, back, shoulder, and snout windows and to apply prompt strategies during animal transport [100]. To evaluate the thermal response of animals to different settings, IRT is used in specific anatomical regions or thermal windows where high densities of blood capillaries and arteriovenous anastomoses can be found [94,101]. Mota Rojas et al. [25] mention that in large ruminants, thermal windows such as the ocular, nasal, udder, and vulvar windows are relevant, and that these windows can be classified into central (e.g., ocular, auricular, and thoracic) and peripheral regions (e.g., forelimbs, hindlimbs, and tail). This classification is relevant because thermal imagining highly depends on the anatomical site and its hemodynamic interpretation is closely related. For example, when animals are exposed to a stressor, the blood flow shifts from peripheral regions to central sites, resulting in increased surface temperatures in the ocular/auricular windows while a transient vasoconstriction would reduce the temperature of the hind/forelimbs. Similarly, when exposed to heat stress, the surface temperature of all regions might be due to the vasodilation to increase heat dissipation [44].

In this sense, Barros et al. [102] used IRT on 10 Murrah buffalo bulls, in whom the surface temperatures of the orbital area, left flank, right flank, and scrotum were positively correlated with the THI (r = 0.72, r = 0.77, r = 0.75, and r = 0.41; *p* < 0.0001). This indicates that, if the THI increases, the surface temperature of the buffaloes will also increase. This could serve as a non-invasive method to identify the potential increases in the THI and develop strategies to prevent heat stress. On the other hand, only the orbital temperature was correlated with the RT (r = 0.58, *p* < 0.0001). Brcko et al. [103] found similar results. These authors concluded that the ocular thermal window is positively correlated with the RT. This implies that this anatomical region would be the most useful to determine the thermal stability of the water buffalo.

Regarding the auricular region, Sevegnani et al. [104] evaluated the thermoregulatory response in fifteen dairy Murrah buffaloes in the pre-milking and post-milking periods. They observed that the ear surface temperature, neck temperature, forehead temperature, and back shank temperature increased by 5–12 °C during post-milking. However, the surface temperature in regions such as the flanks and neck were mainly influenced by environmental conditions such as the THI and air temperature. This can lead us to question the usefulness of evaluating the temperature in these regions that are susceptible to environmental changes, contrarily to central windows such as the ocular temperature.

The nasal region has been proposed as an alternative to evaluate the RR of ruminants in response to heat stress. A review made by Idris et al. [105] mentions that, during heat stress, tachypnea is used as an early indicator of thermal stress because a change in the RR can dissipate 30% of the total heat, and is followed by sweating and panting [106,107]. Hahn et al. [107] reported that an increase of 1 °C above the thermal comfort threshold in large ruminants (21.3 °C) increases the RR by approximately 4.3 bpm above the reference value. In this regard, Stewart et al. [108] evaluated the use of IRT and accelerometers in 22 cows (Holstein Friesan and Holstein Friesan x Jersey breed) aged 5.1 years that were exposed to a startling event. The authors found that the evaluation of the RR through IRT and the direct observation of the flank movements in the thoracic region did not present a significant difference, registering an average difference of −0.01 ± 0.87. In addition, the thermal response of the nasal region reliably predicted the HR variability and change in the RR, which would reaffirm the idea that this thermal window could help to assess this physiological parameter. Similarly, Lowe et al. [109] evaluated the thermal fluctuations of the nasal region in five 27-day Hereford calves, finding that the thermal response had a high correlation with the flank movements (r = 0.93), which supports the validation of this thermal window as a non-invasive method to assess the RR. Moreover, the implementation of automated imagining by placing automated thermal cameras in calf feeders to assess the thermal response in this species in real time has increased the accuracy of IRT by 76% [110].

Other regions such as the limbs and horns have also been proposed as thermal windows that are sensitive to heat loss, and examining these regions might serve as a method to recognize hyperthermia or hypothermia processes [111]. For example, Napolitano et al. [14] reported in 109 newborn water buffaloes that the surface temperature in the pelvic limb was 5 °C lower compared to other anatomical regions such as the lacrimal caruncle, periocular, dorsum, or auditory canal during the first five days post calving. Hypothermia is an event that can be present in newborns from different species, as shown in newborn puppies, in whom hypothermia leads to peripheral vasoconstriction of the dorsal metatarsal artery to prevent heat loss [112,113]; this is a similar physiological response as the one observed in buffaloes and can be indirectly evaluated through IRT. However, up to now, the association between thermal stress and these thermal windows has not been clearly established.

Likewise, previous data regarding the horns have reported that these structures participate in thermoregulation [114]. Rodríguez-González et al. [55] observed that the temperature in the frontoparietal region of water buffaloes was at least 7 °C higher after a short period of transport. This could suggest the role of this region when buffaloes are exposed to high temperatures and the importance of blood flow in this area (irrigated by the superficial temporal artery) [114]. Moreover, when considering that this region and the horns are close, this might suggest another thermoregulatory mechanism to lose heat through thinner keratin sheaths [115,116]. Therefore, IRT is proposed as a tool to assess the thermostability of water buffaloes under thermal stress. However, it is important to consider that the response can differ according to the anatomical region, since vasodilation or vasoconstriction dictates the amount of radiated heat that can be detected in the dermal surface, and this can differ in the central and peripheral regions. Furthermore, other environmental factors such as solar radiation, airspeed, and the humidity of the skin might also influence the thermal assessment. For example, the wind speed can decrease the actual temperature of a surface [117], while humidity in the skin might result in cold temperatures (an average of −2 °C) due to the wetness regardless of the actual temperature [118].

## 7. Future Directions

The increasing interest in buffalo farming and the effect that GW has on the thermoregulatory mechanisms of the species needs to address the current improvements that can be made to buffalo farm facilities such as the implementation of shaded areas, sprinklers, fans, and ponds. It is also suggested to investigate the impact that these resources can have on meat-producing water buffaloes since most of the current literature is focused on dairy buffaloes [39,119]. Furthermore, adopting real-time monitoring systems (e.g., automated IRT in animals’ feeders) could alert farmers when microclimate parameters exceed the recommended values or are prone to cause heat stress, triggering physiological alterations in animals. Studies applying precision livestock farming such as sensors, detectors, and cameras, among others, are examples of how the automatic monitoring of animals’ health and climate variables could be considered as a future approach and opportunity to face GW [120].

Likewise, there is an area of opportunity to evaluate the effectiveness of water buffaloes’ thermoregulatory behaviors such as seeking natural and artificial shade, as well as wallowing, through the adaptation of ethograms made exclusively for the species or even automated systems to track and record animal behavior and/or activity. Nonetheless, the influence of individual traits such age, sex, breed, and even their physiological status (e.g., pregnant animals and newborns) needs to be determined [44].

In this sense, recent studies have shown a potential sex-related difference when considering thermoregulatory mechanisms in mammals, where females tend to have a body temperature that is 0.2 and 0.5 °C higher than males, which is probably due to the ovarian cycle and other hormonal changes related to progesterone or estrogens [121]. Although this has not been reported in water buffaloes, it is important to highlight these differences to acknowledge that facilities for males and females might need to be different or adapted to promote thermostability according to the sex. Moreover, the effect of different buffalo breeds and their thermoregulatory response to heat stress might also need studies simultaneously comparing different breeds to conclude if individual variation or genetic adaptability could influence the thermal response of buffaloes.

Therefore, future research needs to focus on individual differences regarding the physiological and behavioral mechanisms of thermoregulation. This could help to propose intervention protocols to monitor and minimize the effect of heat stress. Additionally, future cost-effective strategies to face GW by genetically selecting heat-tolerant buffalo breeds to mitigate or reduce the adverse physiological, productive, and economical effects of heat stress on buffalo farming need further analysis [8].

## 8. Conclusions

GW is a current challenge for water buffalo farms due to the consequences that heat stress has on the species, particularly because buffaloes are reared in tropical and humid areas with hot climates that are prone to increase in temperature as climate change progresses.

While buffaloes have certain morphoanatomical traits that can make them susceptible to heat stress (e.g., dark skin), they also have anatomical advantages such as a thick epidermis, high density of blood vessels, sparsely distributed hair, and larger sweat glands than Bos cattle. Moreover, behaviors such as wallowing help to reduce their core temperature and help them return to a thermoneutral state when challenged by GW.

Understanding that heat stress causes a shift in energy mobilization to restore homeostasis and prevent hyperthermia is important because most of the physiological, endocrine, and behavioral modifications that buffaloes use to return to a thermoneutral state could be diminished by providing an appropriate environment. In this sense, the use of fans, sprinklers, foggers, and natural sources of water (e.g., ponds, pools, and mud puddles) helps them to lose heat via evaporation. Moreover, providing water satisfies the biological need of water buffaloes to wallow, improving both their thermal state and welfare.

## Figures and Tables

**Table 1:** Physiological modifications in water buffalo due to the roofing material.

Evaluated Material	Preferred Material	Physiological Response	THI	Region	Reference
G1: Corrugated iron roof G2: Corrugated iron roof and shade cloth	G2	Male swamp buffaloes in G2 had lower RT (39.14 ± 0.07 °C vs. 40.00 ± 0. 10 °C) and cortisol values (2.14 ± 0.24 ng/mL vs. 3.38 ± 0.37 ng/mL).	91.95 vs. 93.66	Chainat Province, Thailand	[90]
G1: Corrugated asbestos roof G2: Corrugated asbestos roof painted white on upper side G3: Corrugated asbestos roof with expanded polyethylene (EPE) G4: Corrugated asbestos roof painted white on upper side and EPE sheet on lower side	G4	Murrah buffalo heifers had RR of 26.60 ± 0.34 counts/min, pulse rate of 59.72 ± 0.74 counts/min, and RT of 38.36 ± 17.73 °C.	G1: 83.55 G2: 83.18 G3: 82.73 G4: 82.03	Hisar, India	[91,92]
G1: Asbestos roof G2: Asbestos roof with bamboo ceiling G3: Thatch roof	G2 and G3	G2 buffaloes had lower pulse rate, glucose, and serum proteins. G3 buffaloes had lower RT and RR.	Not available	Assam, India	[93]

RT: rectal temperature; RR: respiratory rate.

## Data Availability

Not applicable.
